# Plasminogen activator inhibitor-2 (PAI-2) overexpression supports bladder cancer development in PAI-1 knockout mice in *N*-butyl-*N*- (4-hydroxybutyl)-nitrosamine- induced bladder cancer mouse model

**DOI:** 10.1186/s12967-020-02239-6

**Published:** 2020-02-05

**Authors:** Hideki Furuya, Kazukuni Hayashi, Yoshiko Shimizu, Nari Kim, Yutaro Tsukikawa, Runpu Chen, Yijun Sun, Owen T. M. Chan, Ian Pagano, Rafael Peres, Kanani Hokutan, Fumie Igari, Keith S. Chan, Charles J. Rosser

**Affiliations:** 1 Clinical & Translational Research Program, University of Hawaii Cancer Center, Honolulu, HI 96813 USA; 2 Department of Molecular Biosciences and Bioengineering, University of Hawaii at Manoa, Honolulu, HI 96822 USA; 3 Department of Surgery, Samuel Oschin Comprehensive Cancer Institute, Cedars-Sinai Medical Center, Davis Research Building, 110 N. George Burns Road, Los Angeles, CA 90048 USA; 4 Department of Pathology and Laboratory Medicine, Samuel Oschin Comprehensive Cancer Institute, Cedars-Sinai Medical Center, Los Angeles, CA 90048 USA; 5 Department of Computer Science and Engineering, State University of New York at Buffalo, Buffalo, NY 14203 USA; 6 Department of Microbiology and Immunology, State University of New York at Buffalo, Buffalo, NY 14203 USA; 7 Cancer Prevention in Pacific Program, University of Hawaii Cancer Center, Honolulu, HI 96813 USA

**Keywords:** Plasminogen activator inhibitor-1 (PAI-1) knockout mouse, PAI-1, PAI-2, Serine protease inhibitors

## Abstract

**Background:**

Accumulating evidence suggests that plasminogen activator inhibitor-1 (PAI-1) plays an important role in bladder tumorigenesis by regulating cell cycle. However, it remains unclear whether and how inhibition of PAI-1 suppresses bladder tumorigenesis.

**Methods:**

To elucidate the therapeutic effect of PAI-1 inhibition, we tested its tumorigenicity in PAI-1 knockout (KO) mice exposed to a known bladder carcinogen.

**Results:**

PAI-1 deficiency did not inhibit carcinogen-induced bladder cancer in mice although carcinogen-exposed wild type mice significantly increased PAI-1 levels in bladder tissue, plasma and urine. We found that PAI-1 KO mice exposed to carcinogen tended to upregulate protein C inhibitor (PAI-3), urokinase-type plasminogen activator (uPA) and tissue-type PA (tPA), and significantly increased PAI-2, suggesting a potential compensatory function of these molecules when PAI-1 is abrogated. Subsequent studies employing gene expression microarray using mouse bladder tissues followed by post hoc bioinformatics analysis and validation experiments by qPCR and IHC demonstrated that SERPING1 is further downregulated in PAI-1 KO mice exposed to BBN, suggesting that SERPING1 as a potential missing factor that regulate PAI-2 overexpression (compensation pathway).

**Conclusions:**

These results indicate that serpin compensation pathway, specifically PAI-2 overexpression in this model, supports bladder cancer development when oncoprotein PAI-1 is deleted. Further investigations into PAI-1 are necessary in order to identify true potential targets for bladder cancer therapy.

## Background

Cancer of urinary bladder is the fifth most common cancer with an estimated 80,470 newly diagnosed cases and an estimated 17,670 deaths in 2019 in the US [1]. Despite improvement of diagnosis and treatment of bladder cancer (BCa), the death rates of BCa have been stable over the past two decades [2, 3].

Plasminogen activator inhibitor-1 (PAI-1) is an endogenous inhibitor of urokinase-type plasminogen activator (uPA). The canonical function of PAI-1 is inhibition of tissue plasminogen activator (tPA) and uPA to maintain clot formation and thus plays a major role in non-neoplastic disorders, such as deep vein thrombosis, myocardial infarction, atherosclerosis, and stroke [4, 5]. PAI-1 expression is regulated by many intrinsic factors (e.g, cytokines and growth factors) and extrinsic factors (e.g, cellular stress) [6]. Based on our previous studies, we have identified PAI-1 to be one in a panel of 10 urine-based diagnostic biomarkers for the detection of BCa [7]. We have reported that mRNA and protein levels of PAI-1 are elevated in voided urine from patients with BCa [8–13]. In addition, we confirmed that the protein expression of PAI-1 was increased in actual bladder tumors, compared to benign controls and specifically PAI-1 expression levels were higher in muscle invasive bladder cancer (MIBC) compared with non-muscle invasive bladder cancer (NMIBC) [14, 15].

Recent studies regard PAI-1 as a pleiotropic factor exerting diverse cellular effects, many potentially related to tumorigenesis, including both pro- and anti-tumoral effects [16, 17]. For example, overexpression of PAI-1 inhibited tumor growth in a prostate cancer xenograft model [16], while knockdown of PAI-1 inhibited tumor growth and angiogenesis in fibrosarcoma, colon, lung and breast cancer xenograft models using PAI-1 null nude mice [17]. We also found that genetic and pharmacologic inhibition of PAI-1 in BCa xenografts using PAI-1^+/+^ nude mice significantly decreased tumor size while overexpression of PAI-1 in xenograft resulted in a substantial increase in tumor size [14, 18]. Contrary to xenograft results, PAI-1 knockout mice in carcinogenesis models failed to demonstrate any effect of PAI-1 inhibition on tumor incidence, growth and metastasis. The first report of PAI-1 KO mice in a carcinogenesis model was published by Almholt et al. [19]. In this report, PAI-1 deficiency had no effect on the development and metastasis of mammary tumors induced by polyoma virus middle T antigen (PyVT). Similarly, PAI-1 KO mice did not affect the development of colon cancer, ocular tumors and skin cancer [20–22]. It is still unclear whether this is due to the functions of compensatory serine protease inhibitors (serpins), including PAI-2, protein C inhibitor (PAI-3), protease nexin-1 or maspin. It might be possible that PAI-1 does not play an important role in those cancers. Thus, the role of PAI-1 in tumorigenesis and cellular growth is complicated, varying with experimental design and its cellular origin, so more mechanistic studies as well as preclinical studies are required to elucidate the exact role PAI-1 plays in human cancers.

In the current study, we found that PAI-1 KO mice treated with a BCa carcinogen developed BCa including carcinoma in situ (CIS), NMIBC and MIBC with similar incidences as wild type mice by overexpressing PAI-2 that may compensate for the PAI-1 deficiency. Global gene expression microarray and bioinformatics post hoc analysis identified 18 genes, which may be involved in the PAI-2 overexpression, compensatory pathway. IHC analysis revealed that SERPING1 was downregulated only in PAI-1 KO mice by BCa carcinogen treatment. We then discuss the complexity of PAI-1/PAI-2 compensatory pathway in BCa.

## Methods

### Animals, reagents, and tumor model

Mice were housed and handled in the laboratory animal resources facilities at the University of Hawaii (UH). Mice were maintained under controlled conditions of humidity (50 ± 10%), light (12-h light–dark cycle) and temperature (23 ± 2 °C). All mouse experiments were approved by the institutional animal care and use committees at the UH (IACUC # 14-1937). PAI-1 homozygous knockout (KO) mice (mixed background strains derived from C57BL/6 X 129, stock # 002507) were purchased from Jackson Laboratories (Bar Harbor, ME). Based on the suggestion from Jackson Laboratories, we employed C57BL/6 mice as a control. The genotypes of the PAI-1 KO mice were determined by polymerase chain reaction (PCR) analysis of genomic DNA isolated from tail biopsies according to the manufacturer’s protocol. *N*-butyl-*N*-(4-hydroxybutyl) nitrosamine (BBN) was purchased from TCI America (Portland, OR). We employed male and female mice in the same ratio. PAI-1 KO mice and wild-type C57BL/6 wild type (WT) mice at 6 to 8 weeks of age were treated with 0.05% BBN in drinking water continuously for 20 weeks to induce the formation of BCa, which is a well-established mouse carcinogen-induced BCa model [23]. In addition, previous study has shown that PAI-1 is overexpressed in BBN-induced bladder tumor tissues [24, 25]. Mice were weighed weekly. One day before euthanasia, mice were housed in metabolic cages for urine collection. Mice from each group were euthanized in weeks 8 (5 mice/group), 12 (5 mice/group), 16 (5 mice/group), and 20 (20 mice/group) of the experiment (Additional file 1: Fig. S1).

### Histopathology of tumor sections

Resected bladders were initially weighed then each bladder was filled with 100 μl of 10% neutral buffered formalin. The bladder necks were ligated and the entire specimens placed in 10% neutral buffered formalin. Bladders in formalin were embedded in paraffin, sectioned (5 μm) and placed on Superfrost plus Micro slides (Fisher Scientific, Pittsburgh, PA). Deparaffinized sections from each mouse were subjected to hematoxylin and eosin stain for histological evaluation. All samples were assessed by a board-certified pathologist (OC).

### ELISA

Levels of urinary PAI-1 were determined using a commercial ELISA kit (cat. log # MPAIKT-TOT; Molecular Innovations, MI, USA) according to manufacturer’s instruction. The assay was validated by demonstrating (a) undetectable PAI-1 in plasma from PAI-1 null mice and (b) parallel dilution curves for standard PAI-1 and samples from C57BL/6 mice with high endogenous PAI-1.

### Real-time RT-PCR

RNA was extracted from frozen tissues as well as formalin-fixed and paraffin-embedded (FFPE) tissues using RNeasy Mini Kit (Qiagen, Valencia, CA) and RNeasy FFPE Kit (Qiagen), respectively, according to the manufacturer’s instructions. cDNA was synthesized using qScript cDNA SuperMix (Quanta Biosciences, Gaithersburg, MD). Real-time PCR was performed with MyiQ2 Two-Color Real-Time PCR Detection System (Bio-Rad). The standard real-time PCR reaction volume was 20 μl, and consisted of 10 μl of PerfeCTa SYBR Green FastMix (Quanta Biosciences), 7 μl RNAse-free H_2_O, 1 μl forward primer (final concentration of 1 μM), 1 μl reverse primer (1 μM) and 1 μl cDNA (0.5 ng/μl). All reactions were performed in triplicate. Primer sets can be found in Additional file 1: Table S1 and S2. The copy numbers of mRNA were calculated after normalization to β-actin using absolute quantification.

### Gene expression microarray analysis

Total RNA was extracted from frozen tissues at week 20. RNA purity and integrity were evaluated by ND-1000 Spectrophotometer (NanoDrop, Wilmington, USA), and Agilent 2100 Bioanalyzer (Agilent Technologies, Palo Alto, USA). The Affymetrix Whole transcript Expression array process was executed according to the manufacturer’s protocol (GeneChip Whole Transcript PLUS reagent Kit). Briefly, cDNA was synthesized using the GeneChip WT (Whole Transcript) Amplification kit as described by the manufacturer. The sense cDNA was then fragmented and biotin-labeled with TdT (terminal deoxynucleotidyl transferase) using the GeneChip WT Terminal labeling kit. Approximately 5.5 μg of labeled DNA target was hybridized to the Affymetrix GeneChip Mouse Array at 45 °C for 16 h. Hybridized arrays were washed and stained on a GeneChip Fluidics Station 450 and scanned on a GCS3000 Scanner (Affymetrix). Signal values were computed using the Affymetrix^®^ GeneChip™ Command Console software. The assay was performed by the Macrogen (Korea).

### Statistical analysis

Experimental data were expressed as means with SD. All statistical analyses were conducted using the Student *t* test for comparing means of two groups and one-way analysis of variance (ANOVA) with post hoc Tukey test when comparing more than two groups. A *P* value of < 0.05 was considered significant. All statistical analyses and figures were carried out using GraphPad Prism software 7.0 (GraphPad Software, Inc.).

Gene expression data were summarized and normalized with robust multi-average (RMA) method implemented by Affymetrix^®^ Power Tools (APT). We exported the result with gene level RMA analysis and performed the differentially expressed gene (DEG) analysis. Statistical significance of the expression data was determined via fold change. For a DEG set, a hierarchical cluster analysis was performed using complete linkage and Euclidean distance as a measure of similarity. Gene-Enrichment and Functional Annotation analysis for significant probe list was performed using Gene Ontology (www.geneontology.org/) and KEGG(www.genome.jp/kegg/). All data analysis and visualization of differentially expressed genes was conducted using R 3.3.3 (www.r-project.org).

## Results

### PAI-1 levels are upregulated in tissues, plasma and urine by BBN

**Fig. 1:**
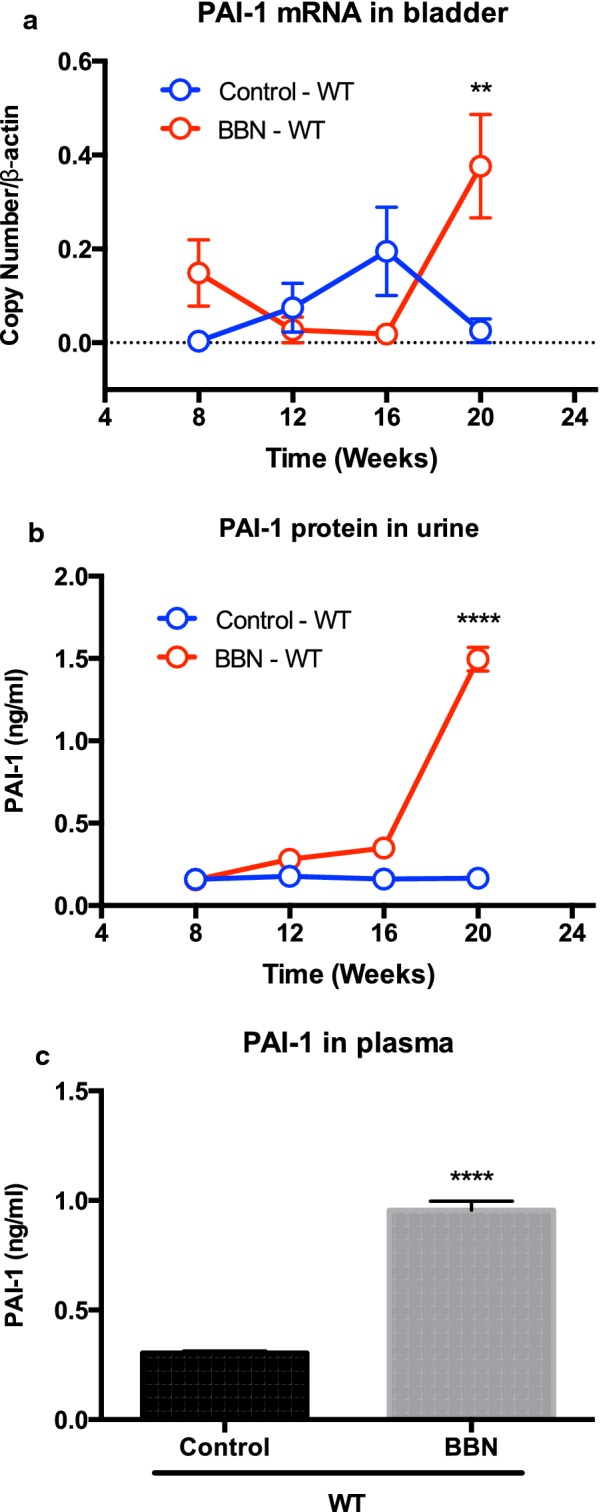
BBN-induced bladder tumorigenesis model. PAI-1 mRNA expression in bladder tissues in WT mice (n = 5 per each time point; ^**^P < 0.01; **a**). PAI-1 protein levels in urine collected from WT mice (n = 5 per each time point; ^****^P < 0.0001; **b**). PAI-1 protein levels in plasma collected from WT mice (n = 5 at week 20; ^****^P < 0.0001; **c**). Bars represent SD

To confirm that PAI-1 is overexpressed by BBN in our animals, we analyzed PAI-1 levels in mouse bladders during BBN exposure using real-time RT-PCR. As we expected, BBN exposure tended to increase PAI-1 expression in week 8, while the BBN exposure tended to reduce PAI-1 expression in weeks 12 and 16 (Fig. 1a). BBN exposure to WT mice significantly increased PAI-1 expression at week 20 when 50% of BBN-exposed mice developed MIBC (*P* < 0.01, Table 1). We also analyzed PAI-1 levels in urine and plasma collected from WT with/without BBN exposure. We found that PAI-1 levels in urine was gradually increased by BBN exposure in a time-dependent manner (Fig. 1b). At week 20 when 50% of BBN-exposed WT mice developed MIBC, the urinary PAI-1 levels in BBN-exposed WT mice was significantly higher than control WT mice (*P* < 0.0001), indicating the strong correlation between PAI-1 levels and bladder tumor grade/stage in this mouse model. Interestingly, PAI-1 levels in plasma was also higher in BBN-exposed WT mice when compared to control WT mice (*P* < 0.0001, Fig. 1c), suggesting that perhaps plasma PAI-1 levels can be a potential cancer biomarker.

**Table 1:** Histological findings in urinary bladder

Strain	BBN treatment	Period (week)	Effective no. of mice	NMIBC		MIBC
				Carcinoma in situ	Ta and T1	
Wild type	No	8	4	0	0	0
		12	4	0	0	0
		16	5	0	0	0
		20	15	0	0	0
PAI-1 KO	No	8	5	0	0	0
		12	4	0	0	0
		16	4	0	0	0
		20	16	0	0	0
Wild type	Yes	8	6	6 (100)^*^	0	0
		12	5	5 (100)	0	0
		16	6	4 (67)	1 (17)	1 (17)
		20	16	6 (38)	2 (13)	8 (50)
PAI-1 KO	Yes	8	5	5 (100)	0	0
		12	4	4 (100)	0	0
		16	5	4 (80)	1 (20)	0
		20	13	2 (15)	5 (38)	6 (46)

*Numbers in parentheses indicate percentage

### Development of CIS, NMIBC and MIBC in PAI-1 KO mice

**Fig. 2:**
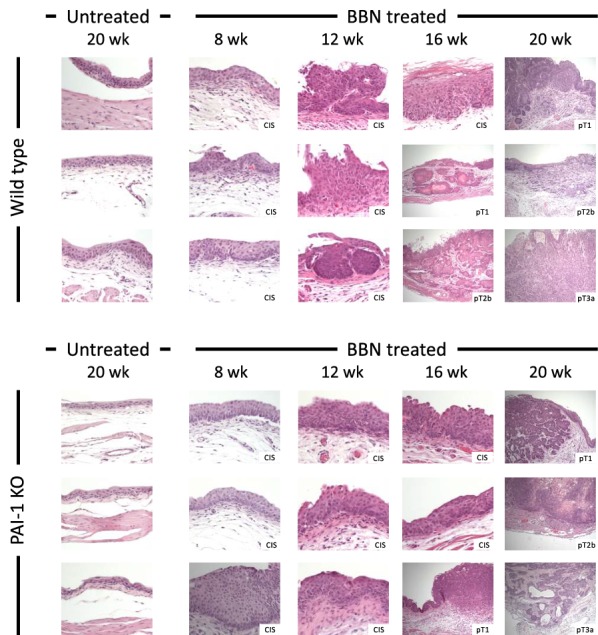
Development of CIS, NMIBC and MIBC in WT and PAI-1 KO mice. H&E staining of bladder urothelium from 3 untreated, aged WT mice (26–28 weeks old); bladder urothelium from 3 WT mice with CIS after 8 weeks of BBN exposure; bladder urothelium from 3 WT mice with CIS after 12 weeks of BBN exposure; bladder urothelium from 3 WT mice with CIS and NMIBC after 16 weeks of BBN exposure; bladder urothelium from 3 WT mice with NMIBC and MIBC after 20 weeks of BBN exposure; 3 untreated, aged PAI-1 KO mice (26–28 weeks old); bladder urothelium from 3 PAI-1 KO mice with CIS after 8 weeks of BBN exposure; bladder urothelium from 3 PAI-1 KO mice with CIS after 12 weeks of BBN exposure; bladder urothelium from 3 PAI-1 KO mice with CIS and NMIBC after 16 weeks of BBN exposure; bladder urothelium from 3 PAI-1 KO mice with NMIBC and MIBC after 20 weeks of BBN exposure. All normal CIS images were captured at 400× magnification and all tumor images were captured at 100× magnification

To determine the influence of PAI-1 deficiency on BBN-induced BCa, we exposed WT and PAI-1 KO mice to 0.05% BBN in drinking water for 20 consecutive weeks. First, the data showed that exposure to BBN was sufficient to induce CIS, NMIBC and MIBC in a time dependent manner (Table 1). For example, BBN exposure to WT mice for 8 and 12 weeks developed CIS in all mice. BBN exposure for 16 weeks developed CIS in 4 of 6 WT mice (67%) and bladder tumors in 2 of 6 WT mice (33%). BBN exposure for 20 weeks developed CIS in 6 of 16 WT (38%) mice and bladder tumors in 10 of 16 WT mice (62%). The results indicate that 0.05% BBN in drinking water developed bladder tumors at a time-dependent rate. As summarized in Table 1, PAI-1 deficiency did not reduce incidence of CIS and bladder tumors. For example, BBN exposure to PAI-1 KO mice for 8 and 12 weeks induced CIS in all mice. Also, BBN exposure to PAI-1 KO mice for 16 and 20 weeks induced bladder tumors in 20% (1/5) and 85% (11/13) of PAI-1 KO mice, respectively. Representative light macroscopic photos of bladder tumors in WT and PAI-1 KO mice are shown in Fig. 2. By histological analysis, we found that 1 of 2 bladder tumors demonstrated NMIBC and another demonstrated MIBC in WT, while one bladder tumor indicated NMIBC in PAI-1 KO mice at week 16. At week 20, 2 of 10 bladder tumors demonstrated NMIBC and 8 of 10 bladder tumors demonstrated MIBC in WT mice, while 5 of 11 bladder tumors demonstrate NMIBC and 6 of 11 bladder tumors demonstrate MIBC in PAI-1 KO mice. However, we observed no histological difference including aggressiveness, invasiveness and inflammation between WT and PAI-1 KO mice (Fig. 2). Taken together, the results indicate that genetic deletion of PAI-1 did not reduce tumor incidence in the BBN-induced bladder tumor model.

### Expression of PAI-2, PAI-3, protease nexin-1, maspin, uPA and tPA in PAI-1 KO and WT bladders

**Fig. 3:**
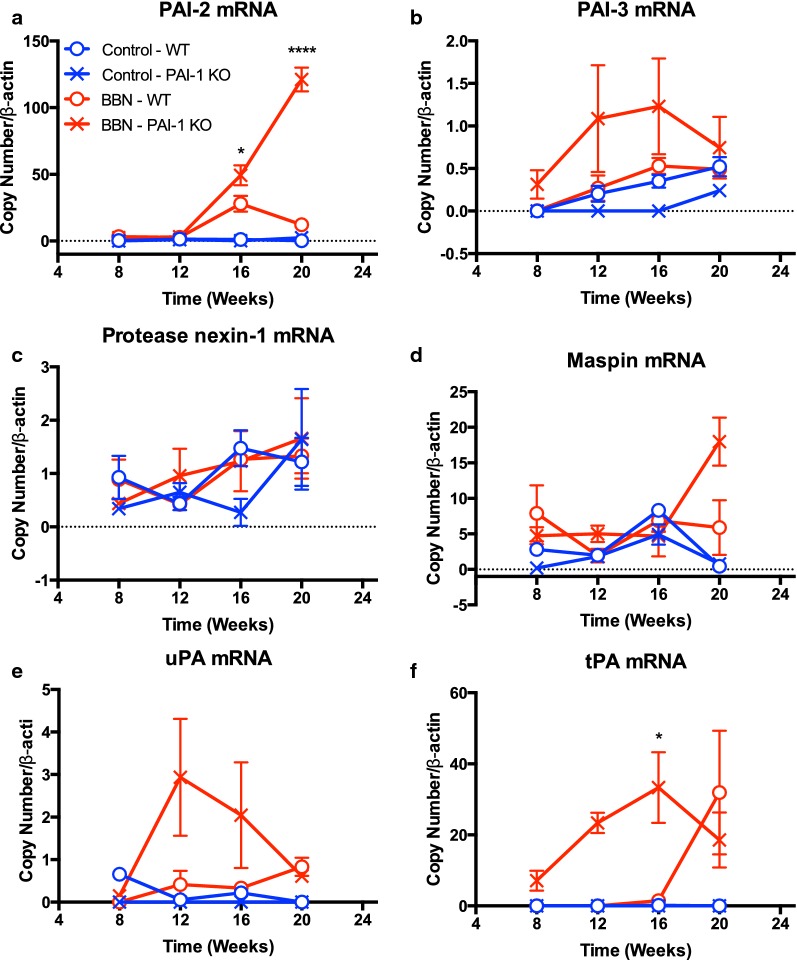
PAI-2, PAI-3, protease nexin-1, maspin, uPA and tPA mRNA levels in bladder tissues. Blue circle, Control-WT; blue cross, Control-PAI-1 KO; red circle, BBN-WT; red cross, BBN-PAI-1 KO. Bars represent SD. *P < 0.05, BBN-WT vs. BBN-PAI-1 KO; ****P < 0.0001, BBN-WT vs. BBN-PAI-1 KO

To determine the mechanisms by which lack of PAI-1 expression showed no effect on BBN-induced BCa development, we analyzed the mRNA levels of PAI-2, PAI-3, protease nexin-1, maspin, uPA and tPA in bladder tissues using real-time RT-PCR (Fig. 3). In WT mice, BBN exposure increased PAI-1 expression at week 16 and 20, and uPA and tPA at week 20 (Fig. 3e and f). Interestingly, PAI-1 deficiency extremely augmented PAI-2 expression induced by BBN exposure at week 16 and PAI-2 expression was further increased at week 20 (Fig. 3a), suggesting that PAI-2 may compensate for an a low or absent PAI-1. On the other hand, we observed different responses in PAI-3, uPA and tPA expression in PAI-1 KO mice. Although their expression levels were slightly affected by BBN in WT mice, PAI-1 deficiency significantly induced high expression levels of PAI-3, uPA and tPA at weeks 12 and 16, and then their levels went back to the baseline levels of WT mice (Fig. 3b, e, f). The results indicate that PAI-1 deficiency changes the balance of serpins, which may be associated with bladder tumorigenesis. There is no difference in protease nexin-1 and maspin expression in PAI-1 KO and WT mice with/without BBN exposure (Fig. 3c, d), indicating that protease nexin-1 and maspin do not play roles in bladder tumorigenesis.

### Effect of PAI-1 knockdown and overexpression in human urothelial cells in vitro

To further investigate the relationships of serpins in PAI-1 KO mice, we genetically manipulated PAI-1 in urothelial cells and analyzed the expression of serpins. We first employed 2 siRNAs for PAI-1 to knockdown PAI-1 expression in UM-UC-3 cells, and then analyzed expression of serpins. As shown in Additional file 2: Fig. S2A, both siRNAs significantly inhibited PAI-1 expression. Interestingly, both siRNAs also downregulated PAI-2 and protease nexin-1 expression when compared to the cells transfected with siRNA for scramble-negative control (SCR) (Additional file 2: Fig. S2A, left). We also employed commercial PAI-1 siRNA. We found that the commercial PAI-1 siRNA also significantly downregulated PAI-2 and protease nexin-1 expression as well as PAI-1 (data not shown). The results from transient knockdown of PAI-1 with siRNA are different from in vivo experiments in that PAI-1 deficiency upregulated PAI-2 but did not change protease nexin-1 levels. This may be due to the difference in existence of PAI-1 between transient knockdown and spontaneous knockout. Therefore, we established stable transfectants carrying SCR and PAI-1 shRNA. Unlike transient transfectants, long-term knockdown of PAI-1 downregulated only protease nexin-1, but not PAI-2 (Additional file 2: Fig. S2A, right). In addition, we overexpressed PAI-1 in urothelial cells with low PAI-1 expression, UROtsa and 5637. Interestingly in both cell lines, forced overexpression of PAI-1 led to upregulation of PAI-3 and maspin (Additional file 2: Fig. S2B). PAI-1 overexpression increased protease nexin-1 expression in UROtsa, while protease nexin-1 expression was reduced in 5637 cells. Thus, the results indicate that regulation of serpins is complicated and not simple as general signaling cascades.

### Global gene expression analysis by microarray, validation by qPCR and IHC

**Fig. 4:**
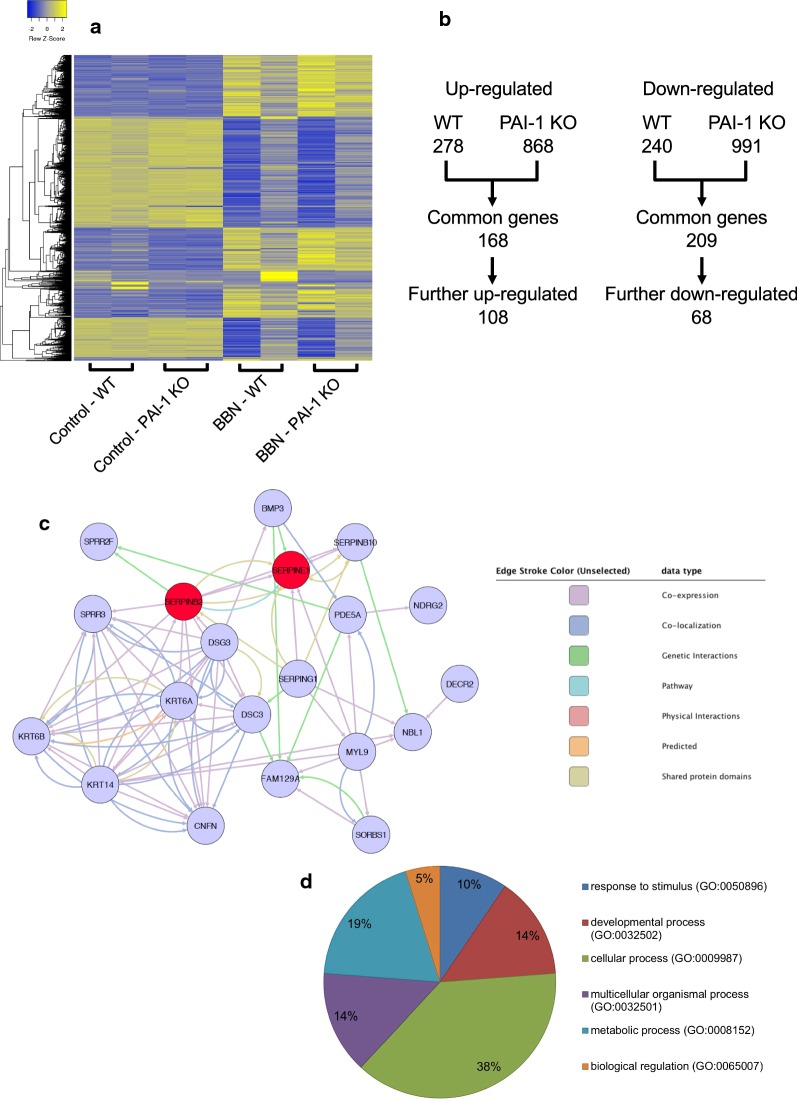
Global gene expression analysis identified key genes that were changed by BBN treatment in PAI-1 KO mice. **a** Whole genome expression profile evaluated by microarray analysis assessed the expression of 41,345 gene transcripts. Gene expression microarray heatmap (n = 2 per group) of dysregulated genes. **b** Strategy for gene selection. Since PAI-2 is upregulated by BBN but PAI-1 deficiency further upregulated PAI-2, we first compare between control and BBN in WT and PAI-1 KO mice, respectively. Then we identified common genes that are found in both WT and PAI-1 KO mice. With the common genes, we selected further up- or down-regulated genes. **c** Gene interaction map with 9 most upregulated and 9 most downregulated genes along with PAI-1 and PAI-2. **d** Pathway analysis of the genes identified in **c**

**Fig. 5:**
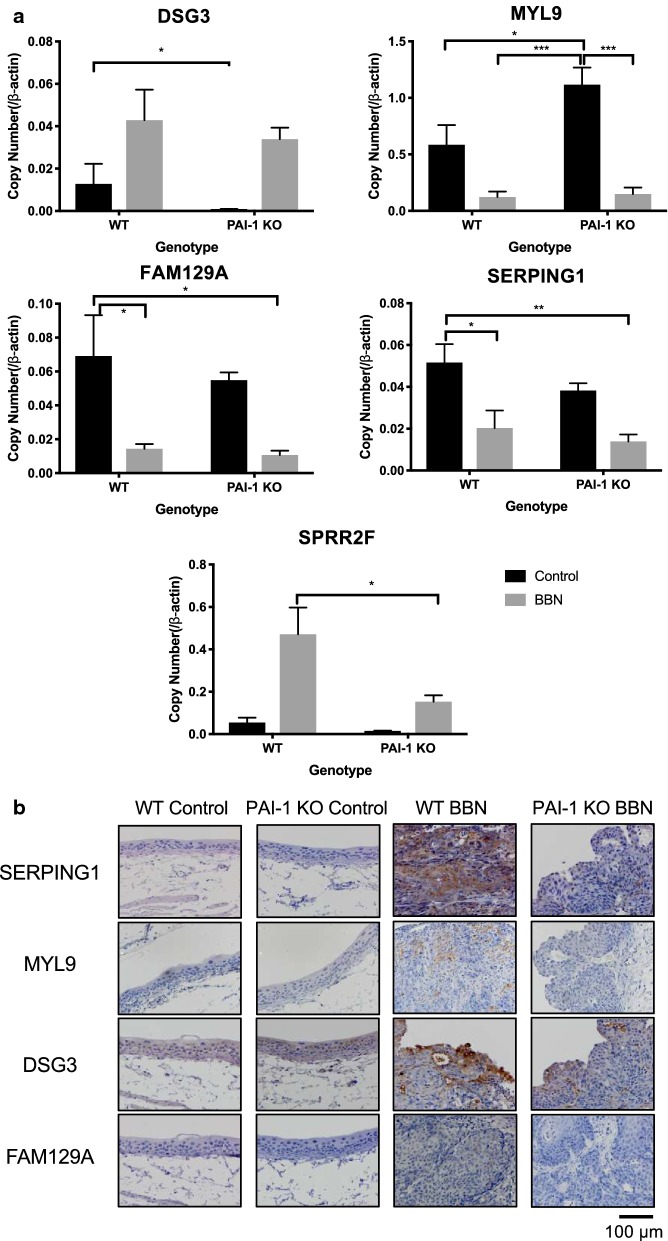
Validation of selected genes by qPCR (**a**) and IHC (**b**). The qPCR data indicates copy number (target gene/β-actin). Bars represent SE. *P < 0.05; **P < 0.01; ***P < 0.001

Because in vitro experiments using cell lines did not replicate the results from the animal experiment, we set out to explore the mechanism by which PAI-1 deficiency along with BBN exposure could induce PAI-2 overexpression leading to bladder tumorigenesis. Using gene expression microarray, we investigated the effect of PAI-1 deficiency and BBN on 41,345 transcripts in BBN-induced mouse bladder tumors (GEO accession number GSE140457). The gene expression profiles of tumors were compared and analyzed with a cutoff of fold changes at 1.5 and P value of 0.05. Since PAI-2 is upregulated by BBN but PAI-1 deficiency further upregulated PAI-2, we first compare between control and BBN in WT and PAI-1 KO mice, respectively. We identified 868 genes upregulated and 991 genes downregulated in bladders from PAI-1 KO mice, while 278 genes upregulated and 240 genes downregulated in bladders from WT mice (Fig. 4a, b). We then identified 168 common genes upregulated and 209 common genes downregulated. With the common genes, we selected further upregulated or downregulated genes; 108 and 68 genes, respectively. The 9 most upregulated and 9 most downregulated genes were selected, and we performed gene interaction map analysis with them (Fig. 4c). With 18 identified genes along with PAI-1 and PAI-2, we performed pathway analysis using PANTHER pathway analysis (http://www.pantherdb.org). The results identified cellular process (DSG3, SPRR3, PDE5A, NDRG2, BMP3, DSC3, SPRR2f) as the highest ranked functional groupings and associated canonical pathways included metabolic process, multicellular organismal process and response to stimulus (Fig. 4d). By qPCR analysis, we validated the concordance with the gene expression microarray in MYL9, FAM129A, SERPING1, DSG3 and SPRR2F (Fig. 5a and Additional file 2: Fig. S3). Since an antibody for mouse SPRR2F was not commercially available, we performed IHC for the remaining 4 targets (MYL9, FAM129A, SERPING1 and DSG3). Interestingly, IHC results for SERPING1 demonstrated statistically significant difference but the results were different from qPCR analysis. In mRNA levels by qPCR, PAI-1 deficiency decreased SERPING1 expression, and BBN treatment further decreased its expression. However, in protein levels by IHC, BBN treatment increased SERPING1 expression, while PAI-1 deficiency tempered this increase (Fig. 5b and Additional file 2: Fig. S4).

## Discussion

In this study, we first showed that BBN-exposure to WT mice increased PAI-1 levels in tissue, plasma and urine. However, we also showed that PAI-1 deficiency did not inhibit BBN-induced bladder tumor development, including CIS, NMIBC and MIBC. This may be due to overexpression of PAI-2, which may compensate for PAI-1 deletion and lead to bladder tumor development. Global gene expression analysis identified MYL9 and SERPING1 as potential downstream target of PAI-1, which may regulate PAI-1/PAI-2 compensatory pathway.

The major finding in this study is the complicated balance of serpins system in bladder tumorigenesis. Based on previous studies including our reports [14, 17, 18, 26–28], we hypothesized that PAI-1 plays an important role in bladder tumor development. However, this study employing PAI-1 KO mice in BBN-induced bladder tumor model demonstrated that PAI-1 deficiency did not inhibit tumor incidence or progression. In fact, previous reports employing PAI-1 KO mice have also failed to demonstrate any effect of PAI-1 deficiency on tumor initiation, growth and metastasis [19, 21, 22, 29]. A recent review article by Placencio et al. hinted that this may be due to the presence of compensatory serpins including PAI-2, PAI-3, protease nexin-1 and maspin [5]. We first analyzed PAI-1 levels in this model. We confirmed that BBN exposure in WT mice induced the process of bladder tumorigenesis in a time-dependent manner accompanying the increase in PAI-1 levels in tissue, plasma and urine. We also found that PAI-1 expression in BBN exposed bladders was significantly higher than normal bladders in 20 weeks. In addition, PAI-1 levels in urine and plasma were also increased in BBN-exposed WT mice. The results from the animal experiments are similar to our previous reports in humans in that PAI-1 expression is higher in bladder tumor tissues than in benign tissue and higher in MIBC than in NMIBC [15, 30].

Notably, we found that levels of PAI-2, PAI-3, uPA and tPA in bladder were modified by PAI-1 deficiency. Several studies demonstrated that PAI-1 deficiency is not compensated by the overexpression of proteins in plasminogen/plasmin system (PAI-2, PAI-3, protease nexin-1, maspin, tPA, uPA and uPAR) [19, 21, 22, 29]. However, since previous studies analyzed the levels after tumors were developed, the effect of PAI-1 deficiency on plasminogen/plasmin system remains unclear. Therefore, we investigated the changes in their expression during the process of bladder tumorigenesis. Interestingly, in contrast to previous studies, PAI-1 KO mice exposed to BBN increased PAI-2, PAI-3, uPA and tPA mRNA levels in their bladder, suggesting that PAI-1 deficiency may be compensated by all or some of these proteins. PAI-2 is expressed in placenta, thus called placental PAI. Its canonical function is the same as PAI-1, a coagulation factor that inactivates uPA and tPA. However, PAI-2 has been reported to have different functions from PAI-1 in cancer. High levels of PAI-1 in tumor promote tumor growth and progression, while high PAI-2 levels decrease tumor growth and metastasis by inhibiting uPA [31]. Crucial structural differences in PAI-2 preclude direct high-affinity binding to vitronectin or members of the LDLR family and hence extracellular PAI-2 does not have the capability to induce these additional cellular responses. On the other hand, PAI-2 has been reported to have an ability to prevent apoptosis. Specifically, PAI-2 has been found in the nucleus, where it may interact with RB leading to the prevention of RB degradation [31]. The findings indicate that PAI-2 function in tumorigenesis is still unclear. PAI-3 is known as protein C inhibitor, which limits the activity of protein C (an anticoagulant). In addition to the canonical function, PAI-3 has been reported to inhibit breast cancer growth and metastasis [32]. However, a recent study demonstrated that PAI-3 inhibits melanoma tumor growth but promotes its tumor metastasis to lung [33]. uPA and tPA are serine proteases, which convert plasminogen (inactive) to plasmin (active). It has been reported that uPA is highly expressed in most solid and hematologic cancers and thus uPA has been thought to be a potential therapeutic target [34]. In fact, genetic and pharmacological inhibition of uPA successfully exhibited slower tumor growth and showed less tumor progression in vivo. The reports suggest that uPA upregulation may be associated with the induction of bladder tumorigenesis in PAI-1 KO mice. However, since uPA was upregulated in weeks 12 and 16 in PAI-1 KO mice when PAI-1 was downregulated in WT mice exposed to BBN, the effect of uPA upregulation on bladder tumorigenesis in this model remains unclear. Taken all together, due to these conflicting results, it remains unclear whether PAI-2, PAI-3, uPA and/or tPA may compensate for a loss of PAI-1 in mouse bladder tumorigenesis. However, because PAI-2 is extremely upregulated by BBN treatment in PAI-1 KO mice, we hypothesize that PAI-2 may compensate for loss of PAI-1 resulting in bladder tumor development. To the best of our knowledge, this is the first report to show the effect of PAI-1 deficiency on the serpins system, specifically regarding a potential function of PAI-2 in tumorigenesis.

Further investigating the mechanism by which PAI-1 KO mice exposed to BBN overexpressed PAI-2, PAI-3, uPA and tPA mRNA levels, we genetically manipulated PAI-1 in urothelial cell lines and analyzed mRNA levels of serpins. Unlike PAI-1 KO mice exposed to BBN, genetic inhibition of PAI-1 in UM-UC-3 cells reduced PAI-2 and protease nexin-1 levels. Based on BLAST search, the siRNAs for PAI-1 are specific to PAI-1 and are not supposed to bind to PAI-2 or protease nexin-1. In addition, we found that forced overexpression of PAI-1 in UROtsa and 5637 cells increased PAI-3 and maspin. These results from in vitro studies are not consistent with the data from our in vivo study, indicating the complicated regulation of serpin network.

To identify the pathway how BBN treatment in PAI-1 KO mice increase PAI-2 expression, we performed global gene expression analysis by microarray using mouse bladder tissues followed by validation experiments by qPCR and IHC. We identified SERPING1 as a potential missing factor that regulate PAI-2 overexpression (compensation pathway). SERPING1 is also known as C1 esterase inhibitor and a protease inhibitor belonging to the serpin superfamily. Previous study has reported that a high-protein diet resulted in decreased SERPING1 expression and increased PAI-1 expression, which may be one of factors of elevated urinary urea [35]. The stress of high urinary urea concentration induced an abnormally activated inflammatory response, cell cycle arrest, apoptosis and pathways in cancer occurred in bladder urothelium. The evidence suggested that high urinary urea concentration caused by high-protein diet might be a potential carcinogenic factor in bladder. Another line of study has shown that decreased expression of SERPING1 can be a biomarker for risk of prostate cancer and prediction of malignant progression [36]. In addition, analyses of serpins in matched tissue and mucus exosomal proteins in chronic rhinosinusitis tissue demonstrated upregulated PAI-1 and PAI-2 along with downregulated SERPING1 [37]. The evidence suggest links between PAI-1, PAI-2 and SERPING1, which may, in our case lead to bladder tumorigenesis (Additional file 3).

## Conclusions

In summary, the present study shows that PAI-1 deficiency does not impair BBN’s ability to induce bladder tumorigenesis. In addition, we demonstrated that PAI-1 KO mice exposed to BBN upregulated serpin expression, specifically PAI-2, when compared to WT mice exposed to BBN. Moreover, we observed that SERPING1 was further downregulated in PAI-1 KO mice exposed to BBN, suggesting that SERPING1 served as a potential missing factor that regulate PAI-2 overexpression (compensation pathway). Taken together, these results indicate that the serpin compensation pathway, specifically PAI-2 overexpression in this model, supports bladder cancer development when oncoprotein PAI-1 is deleted. Further investigations into PAI-1 are necessary in order to identify true potential targets for bladder cancer therapy.

## Supplementary information

**Additional file 1: Figure S1.** Experimental scheme. **Figure S2A.** Effect of PAI-1knockdown by transient and stable transfection on serpins in UM-UC-3cells. **Figure S2B.** Effect of PAI-1 overexpression on serpins in UROtsa and 5637 cells. **Figure S3.** Validation of expression of selected genes by qPCR. **Figure S4.** Protein expression of selected targets evaluated by IHC.

**Additional file 2: Table S1**. Real-time PCR primers for human. **Table S2**. Real-time PCR primers for mouse.

**Additional file 3: Table S3.** Pre-processed results of global gene expression microarray.

## Data Availability

The datasets supporting the conclusions of this article are included within the article, its additional file, and GEO accession number GSE140457.

## Abbreviations

PAI-2: Plasminogen activator inhibitor-2
BBN: *N*-butyl-*N*-(4-hydroxybutyl)-nitrosamine
PAI-1: Plasminogen activator inhibitor-1
KO: Knockout
PAI-3: Protein C inhibitor
uPA: Urokinase-type plasminogen activator
tPA: Tissue-type PA
BCa: Bladder cancer
MIBC: Muscle invasive bladder cancer
NMIBC: Non-muscle invasive bladder cancer
PyVT: Polyoma virus middle T antigen
Serpins: Serine protease inhibitors
CIS: Carcinoma in situ
UH: University of Hawaii
PCR: Polymerase chain reaction
WT: Wild type
FFPE: Formalin-fixed and paraffin-embedded
RMA: Robust multi-average
APT: Affymetrix^®^ Power Tools
DEG: Differentially expressed gene
ANOVA: Analysis of variance
SCR: Scramble-negative control

## References

[1] Siegel RL, Miller KD, Jemal A (2019). Cancer statistics, 2019. CA Cancer J Clin.

[2] SEER stat fact sheets: bladder cancer [http://seer.cancer.gov/statfacts/html/urinb.html].

[3] Siegel RL, Miller KD, Jemal A (2017). Cancer statistics, 2017. CA Cancer J Clin.

[4] Ghosh AK, Vaughan DE (2012). PAI-1 in tissue fibrosis. J Cell Physiol.

[5] Placencio VR, DeClerck YA (2015). Plasminogen activator inhibitor-1 in cancer: rationale and Insight for future therapeutic testing. Cancer Res.

[6] Jankun J, Keck RW, Skrzypczak-Jankun E, Swiercz R (1997). Inhibitors of urokinase reduce size of prostate cancer xenografts in severe combined immunodeficient mice. Cancer Res.

[7] Rosser CJ, Chang M, Dai Y, Ross S, Mengual L, Alcaraz A, Goodison S (2014). Urinary protein biomarker panel for the detection of recurrent bladder cancer. Cancer Epidemiol Biomark Prev.

[8] Urquidi V, Goodison S, Cai Y, Sun Y, Rosser CJ (2012). A candidate molecular biomarker panel for the detection of bladder cancer. Cancer Epidemiol Biomarkers Prev.

[9] Urquidi V, Kim J, Chang M, Dai Y, Rosser CJ, Goodison S (2012). CCL18 in a multiplex urine-based assay for the detection of bladder cancer. PLoS ONE.

[10] Goodison S, Chang M, Dai Y, Urquidi V, Rosser CJ (2012). A multi-analyte assay for the non-invasive detection of bladder cancer. PLoS ONE.

[11] Rosser CJ, Ross S, Chang M, Dai Y, Mengual L, Zhang G, Kim J, Urquidi V, Alcaraz A, Goodison S (2013). Multiplex protein signature for the detection of bladder cancer in voided urine samples. J Urol.

[12] Rosser CJ, Chang M, Dai Y, Ross S, Mengual L, Alacaraz A, Goodison S (2014). Urinary protein biomarker panel for the detection of recurrent bladder cancer. Cancer Epidemiol Biomark Prev.

[13] Chen LM, Chang M, Dau Y, Chai KX, Dyrskjot L, Sanchez-Carbayo M, Szarvas T, Zwarthoff EC, Lokeshwar V, Jeronimo C (2014). External validation of a multiplex urinary protein panel for the detection of bladder cancer in a multicenter cohort. Cancer Epidemiol Biomark Prev.

[14] Giacoia EG, Miyake M, Lawton A, Goodison S, Rosser CJ (2014). PAI-1 Leads to G1-phase cell cycle progression through cyclin D3/CDK4/6 up-regulation. Mol Cancer Res.

[15] Zhang G, Gomes-Giacoia E, Dai Y, Lawton A, Miyake M, Furuya H, Goodison S, Rosser CJ (2014). Validation and clinicopathologic associations of a urine-based bladder cancer biomarker signature. Diagn Pathol.

[16] Chen SC, Henry DO, Reczek PR, Wong MK (2008). Plasminogen activator inhibitor-1 inhibits prostate tumor growth through endothelial apoptosis. Mol Cancer Ther.

[17] Fang H, Placencio VR, DeClerck YA (2012). Protumorigenic activity of plasminogen activator inhibitor-1 through an antiapoptotic function. J Natl Cancer Inst.

[18] Gomes-Giacoia E, Miyake M, Goodison S, Rosser CJ (2013). Targeting plasminogen activator inhibitor-1 inhibits angiogenesis and tumor growth in a human cancer xenograft model. Mol Cancer Ther.

[19] Almholt K, Nielsen BS, Frandsen TL, Brunner N, Dano K, Johnsen M (2003). Metastasis of transgenic breast cancer in plasminogen activator inhibitor-1 gene-deficient mice. Oncogene.

[20] Almholt K, Lund LR, Rygaard J, Nielsen BS, Dano K, Romer J, Johnsen M (2005). Reduced metastasis of transgenic mammary cancer in urokinase-deficient mice. Int J Cancer.

[21] Maillard CM, Bouquet C, Petitjean MM, Mestdagt M, Frau E, Jost M, Masset AM, Opolon PH, Beermann F, Abitbol MM (2008). Reduction of brain metastases in plasminogen activator inhibitor-1-deficient mice with transgenic ocular tumors. Carcinogenesis.

[22] Masset A, Maillard C, Sounni NE, Jacobs N, Bruyere F, Delvenne P, Tacke M, Reinheckel T, Foidart JM, Coussens LM, Noel A (2011). Unimpeded skin carcinogenesis in K14-HPV16 transgenic mice deficient for plasminogen activator inhibitor. Int J Cancer.

[23] Vasconcelos-Nobrega C, Colaco A, Lopes C, Oliveira PA (2012). Review: BBN as an urothelial carcinogen. Vivo.

[24] Williams PD, Lee JK, Theodorescu D (2008). Molecular credentialing of rodent bladder carcinogenesis models. Neoplasia.

[25] Yao R, Lemon WJ, Wang Y, Grubbs CJ, Lubet RA, You M (2004). Altered gene expression profile in mouse bladder cancers induced by hydroxybutyl(butyl)nitrosamine. Neoplasia.

[26] Bajou K, Peng H, Laug WE, Maillard C, Noel A, Foidart JM, Martial JA, DeClerck YA (2008). Plasminogen activator inhibitor-1 protects endothelial cells from FasL-mediated apoptosis. Cancer Cell.

[27] McMahon GA, Petitclerc E, Stefansson S, Smith E, Wong MK, Westrick RJ, Ginsburg D, Brooks PC, Lawrence DA (2001). Plasminogen activator inhibitor-1 regulates tumor growth and angiogenesis. J Biol Chem.

[28] Maillard C, Jost M, Romer MU, Brunner N, Houard X, Lejeune A, Munaut C, Bajou K, Melen L, Dano K (2005). Host plasminogen activator inhibitor-1 promotes human skin carcinoma progression in a stage-dependent manner. Neoplasia.

[29] Fen Li C, Kandel C, Baliko F, Nadesan P, Brunner N, Alman BA (2005). Plasminogen activator inhibitor-1 (PAI-1) modifies the formation of aggressive fibromatosis (desmoid tumor). Oncogene.

[30] Miyake M, Lawton A, Dai Y, Chang M, Mengual L, Alcaraz A, Goodison S, Rosser CJ (2014). Clinical implications in the shift of syndecan-1 expression from the cell membrane to the cytoplasm in bladder cancer. BMC Cancer.

[31] Croucher DR, Saunders DN, Lobov S, Ranson M (2008). Revisiting the biological roles of PAI2 (SERPINB2) in cancer. Nat Rev Cancer.

[32] Asanuma K, Yoshikawa T, Hayashi T, Akita N, Nakagawa N, Hamada Y, Nishioka J, Kamada H, Gabazza EC, Ido M (2007). Protein C inhibitor inhibits breast cancer cell growth, metastasis and angiogenesis independently of its protease inhibitory activity. Int J Cancer.

[33] Akita N, Ma N, Okamoto T, Asanuma K, Yoshida K, Nishioka J, Shimaoka M, Suzuki K, Hayashi T (2015). Host protein C inhibitor inhibits tumor growth, but promotes tumor metastasis, which is closely correlated with hypercoagulability. Thromb Res.

[34] Hildenbrand R, Allgayer H, Marx A, Stroebel P (2010). Modulators of the urokinase-type plasminogen activation system for cancer. Expert Opin Investig Drugs.

[35] Liu M, Li M, Liu J, Wang H, Zhong D, Zhou H, Yang B (2016). Elevated urinary urea by high-protein diet could be one of the inducements of bladder disorders. J Transl Med.

[36] Peng S, Du T, Wu W, Chen X, Lai Y, Zhu D, Wang Q, Ma X, Lin C, Li Z (2018). Decreased expression of serine protease inhibitor family G1 (SERPING1) in prostate cancer can help distinguish high-risk prostate cancer and predicts malignant progression. Urol Oncol.

[37] Mueller SK, Nocera AL, Dillon ST, Libermann TA, Wendler O, Bleier BS (2019). Tissue and exosomal serine protease inhibitors are significantly overexpressed in chronic rhinosinusitis with nasal polyps. Am J Rhinol Allergy.

