# Supplementary material for: Plasminogen activator inhibitor-2 (PAI-2) overexpression supports bladder cancer development in PAI-1 knockout mice in N-butyl-N- (4-hydroxybutyl)-nitrosamine- induced bladder cancer mouse model
Source: J Transl Med. 2020 Feb 5;18:57. doi: 10.1186/s12967-020-02239-6 (PMC7003426; doi:10.1186/s12967-020-02239-6)
Supplement: Supplementary file 1 — Additional file 1: Figure S1. Experimental scheme. Figure S2A. Effect of PAI-1knockdown by transient and stable transfection on serpins in UM-UC-3cells. Figure S2B. Effect of PAI-1 overexpression on serpins in UROtsa and 5637 cells. Figure S3. Validation of expression of selected genes by qPCR. Figure S4. Protein expression of selected targets evaluated by IHC. [file 12967_2020_2239_MOESM1_ESM.pptx]

## Slide 1
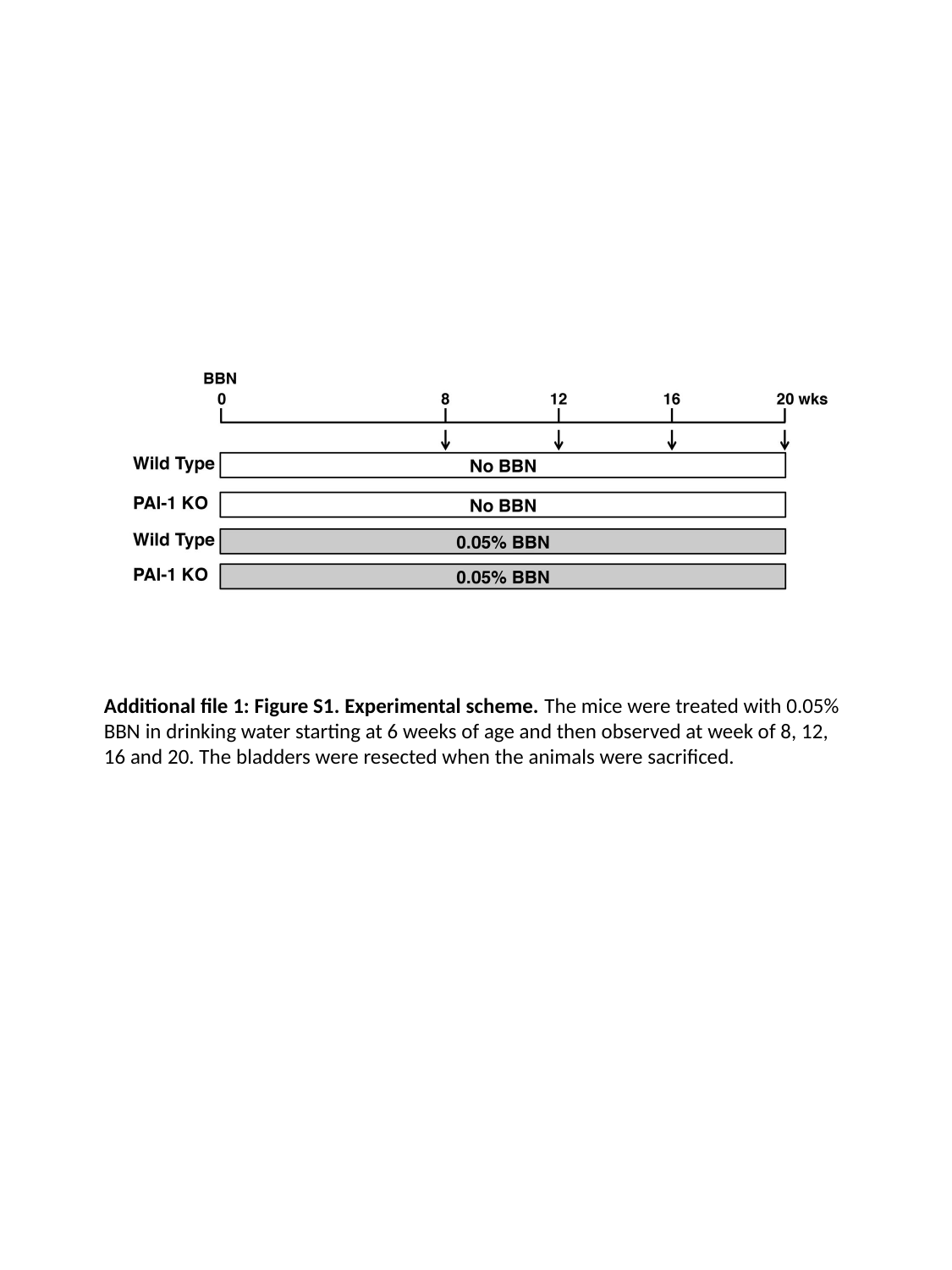

Additional file 1: Figure S1. Experimental scheme. The mice were treated with 0.05% BBN in drinking water starting at 6 weeks of age and then observed at week of 8, 12, 16 and 20. The bladders were resected when the animals were sacrificed.

## Slide 2
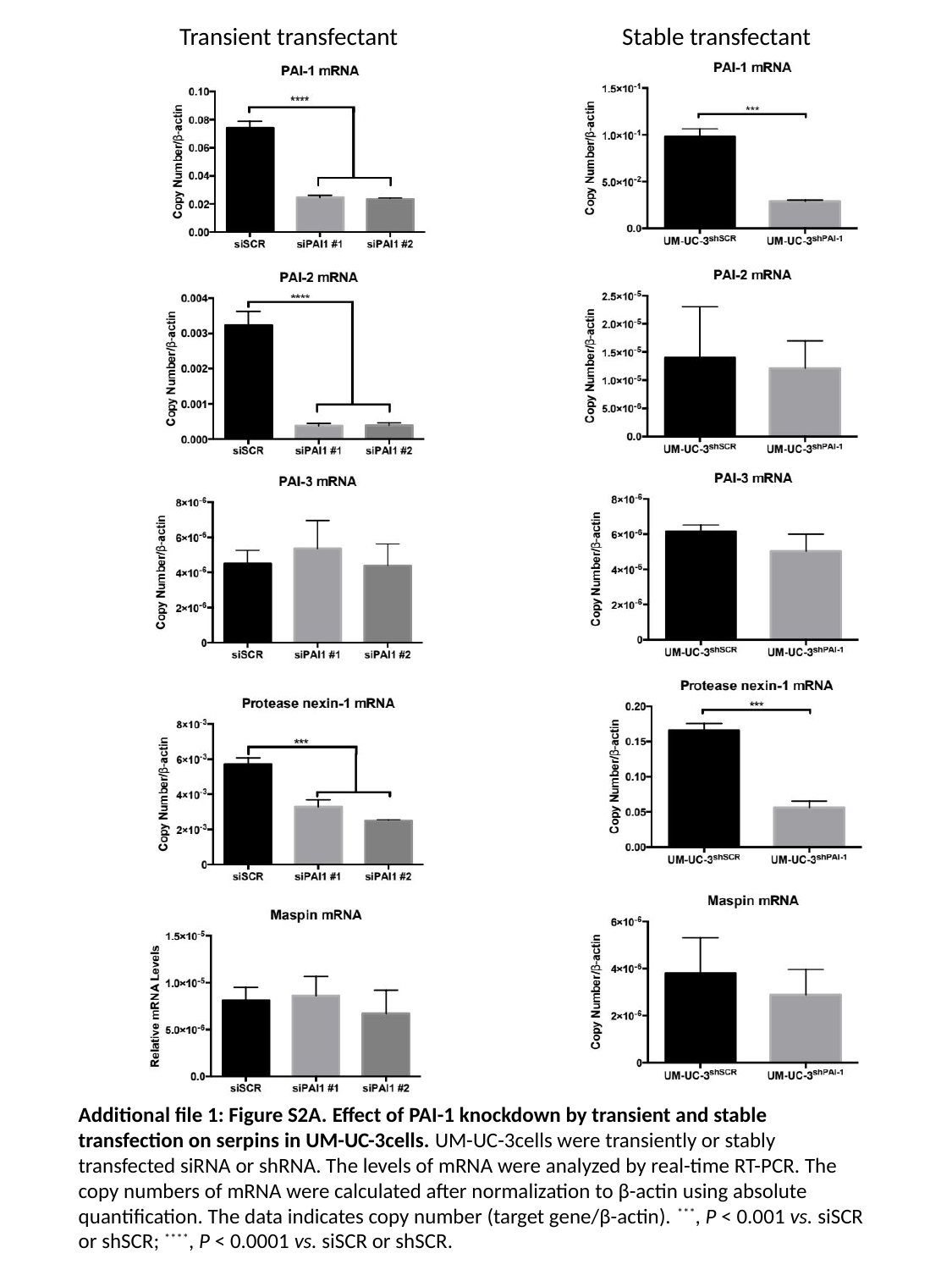

Transient transfectant
Stable transfectant
Additional file 1: Figure S2A. Effect of PAI-1 knockdown by transient and stable transfection on serpins in UM-UC-3cells. UM-UC-3cells were transiently or stably transfected siRNA or shRNA. The levels of mRNA were analyzed by real-time RT-PCR. The copy numbers of mRNA were calculated after normalization to β-actin using absolute quantification. The data indicates copy number (target gene/β-actin). ***, P < 0.001 vs. siSCR or shSCR; ****, P < 0.0001 vs. siSCR or shSCR.

## Slide 3
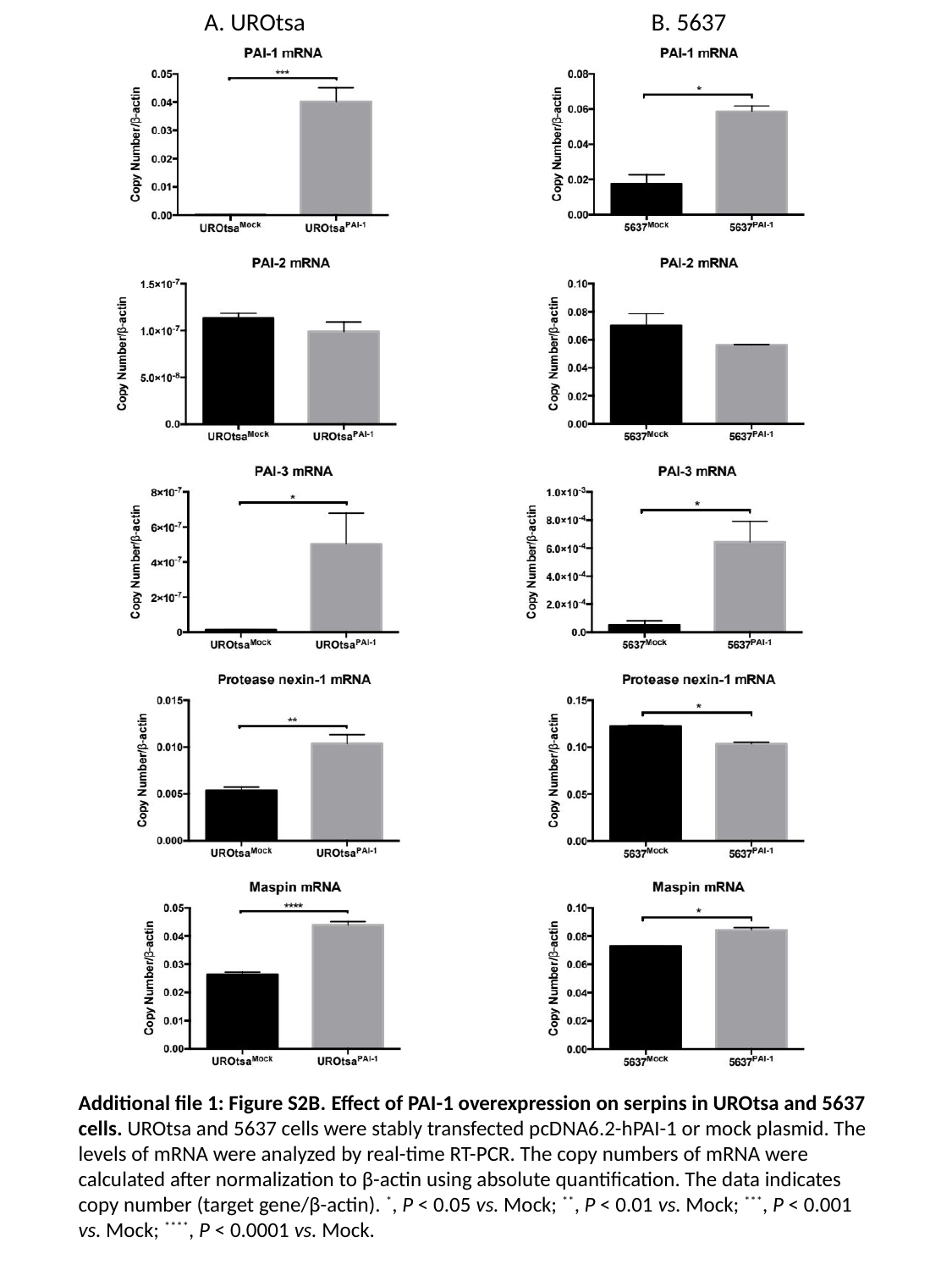

A. UROtsa
B. 5637
Additional file 1: Figure S2B. Effect of PAI-1 overexpression on serpins in UROtsa and 5637 cells. UROtsa and 5637 cells were stably transfected pcDNA6.2-hPAI-1 or mock plasmid. The levels of mRNA were analyzed by real-time RT-PCR. The copy numbers of mRNA were calculated after normalization to β-actin using absolute quantification. The data indicates copy number (target gene/β-actin). *, P < 0.05 vs. Mock; **, P < 0.01 vs. Mock; ***, P < 0.001 vs. Mock; ****, P < 0.0001 vs. Mock.

## Slide 4
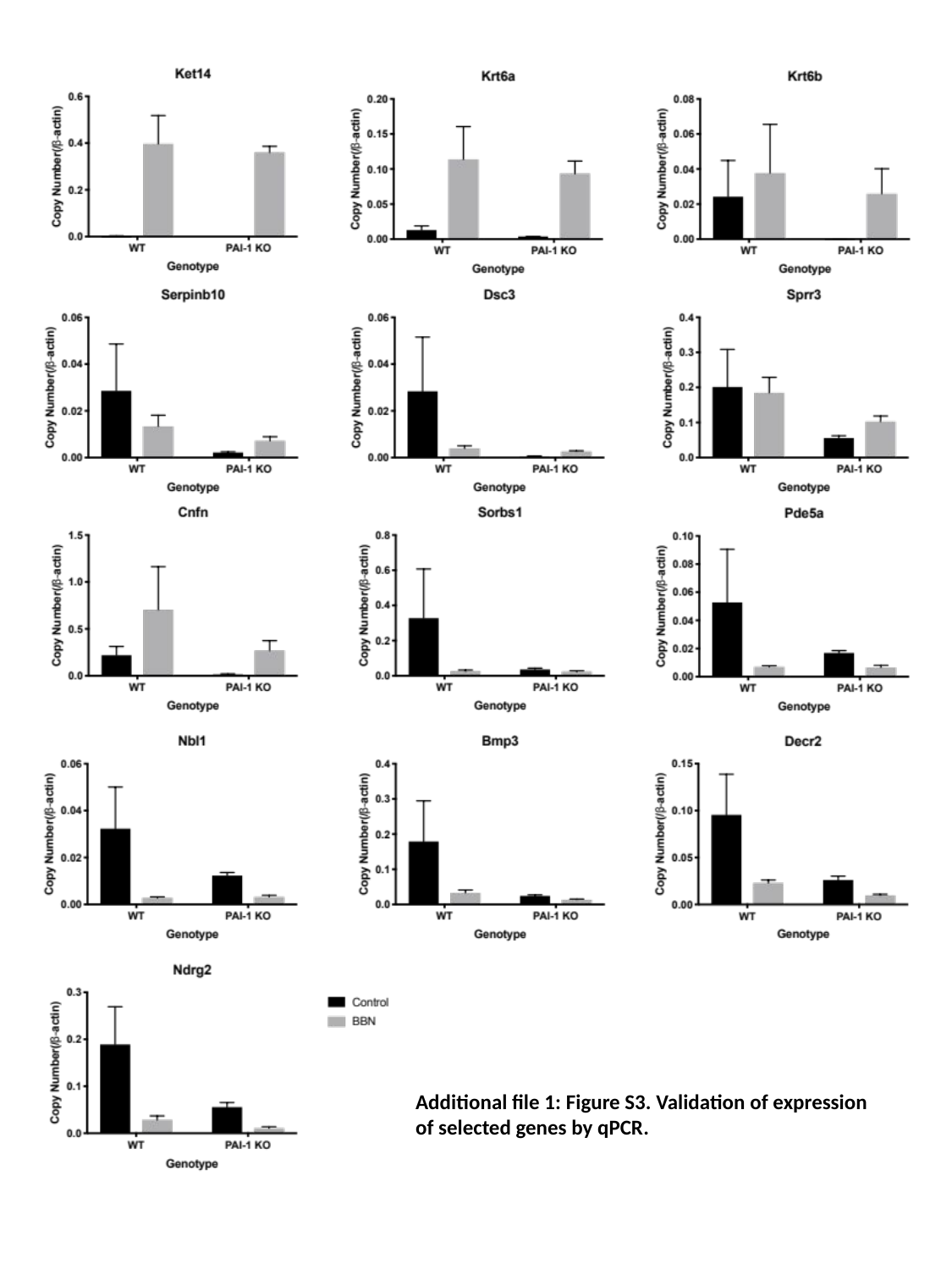

Additional file 1: Figure S3. Validation of expression of selected genes by qPCR.

## Slide 5
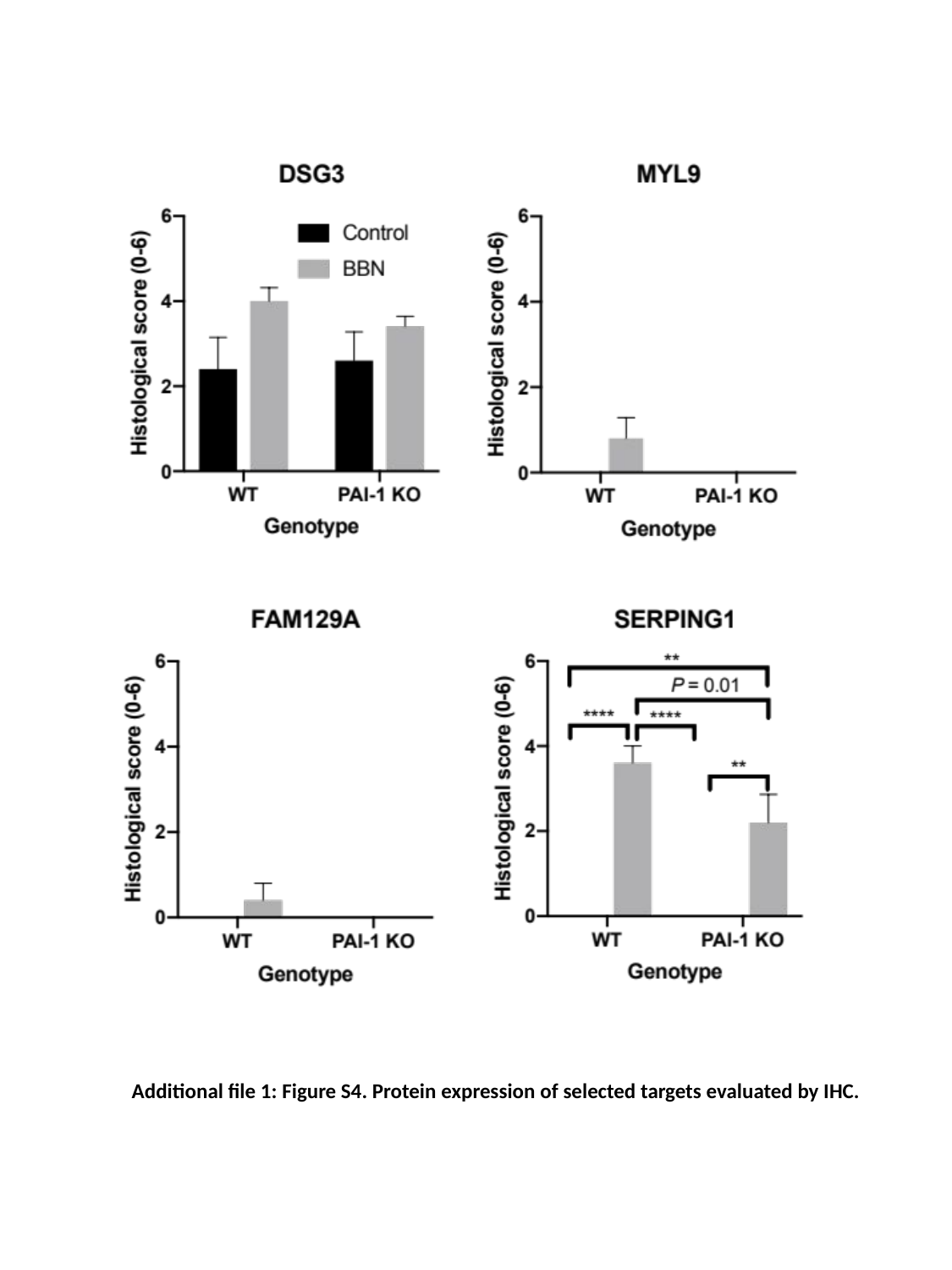

Additional file 1: Figure S4. Protein expression of selected targets evaluated by IHC.
