# Supplementary material for: Plasminogen activator inhibitor-2 (PAI-2) overexpression supports bladder cancer development in PAI-1 knockout mice in N-butyl-N- (4-hydroxybutyl)-nitrosamine- induced bladder cancer mouse model
Source: J Transl Med. 2020 Feb 5;18:57. doi: 10.1186/s12967-020-02239-6 (PMC7003426; doi:10.1186/s12967-020-02239-6)
Supplement: Supplementary file 2 — Additional file 2: Table S1. Real-time PCR primers for human. Table S2. Real-time PCR primers for mouse. [file 12967_2020_2239_MOESM2_ESM.docx]

Table S1 Real-time PCR primers for human

|  | Primer, 5'-3' | |
| --- | --- | --- |
|  | Forward | Reverse |
| PAI-1 | GCCAGTGGAAGACTCCCTTC | GTGCTGCCGTCTGATTTGTG |
| PAI-2 | TCAGAACCCCAGGCAGTAGA | AGTTTGGGATTTTGCCTTTGGT |
| PAI-3 | TGCCACGGCTTTGTTCATTC | CGAGCTGCCTCTTTTTGAACA |
| Protease nexin-1 | GTCCTCGTCAACGCAGTGTA | GTCGACCCACACCGGAAC |
| Maspin | CCACTGGGCAATGTCCTCTT | GAAGAACCTGTCCAATTTCATTTGC |
| β-actin | TCCTCCCTGGAGAAGAGCTA | CCAGACAGCACTGTGTTGGC |

Table S2 Real-time PCR primers for mouse

|  | Primer, 5'-3' | |
| --- | --- | --- |
|  | Forward | Reverse |
| PAI-1 | ATCGCTGCACCCTTTGAGAA | TCCAGAGAGAACTTAGGCAGGA |
| PAI-2 | TTTACAGGCACAAGCAGGAGAT | AATCCCCCTGTGGTGTGTTG |
| PAI-3 | TCTTCAAAGCCAAGTGGCAGA | TCCTTTTGGGGGTCACATGG |
| Protease nexin-1 | GGGTTTGTGGAAGTCTCGGT | GTAGACCCTGAGCGGAACAC |
| Maspin | CTGCAACTCAAGGATGGATGC | GTTTCCTGCTGGGTCCCTTT |
| uPA | ATTCCTGCAAGGGCGATTCT | GGAAGTGTGAGACCCTCGTG |
| tPA | CAGAGATGAGCCAACGCAGA | TTCGCTGCAACTTCGGACAG |
| β-actin | TCCTCCCTGGAGAAGAGCTA | CCAGACAGCACTGTGTTGGC |
| Krt14 | ATCAGTACGAGAAGATGGCG | GGAGCTCAGAAATCTCACTCTTG |
| Krt6a | CAGCATCATTGGAGAGAGGGG | CGAATTCATTCTCTGCTGCTGT |
| Krt6b | ACAAGCGTACAAAAGCAGAGA | AGGAAGTTGATCTCGTCTGCAA |
| Serpinb10 | GGGACTACTGCAGACCAGATG | GCAAGGGTCTGGAAGTCGG |
| Dsc3 | CCCGACTTGGTGAAAAATTGC | TGCTTTTCACTGCAGCAACC |
| Dsg3 | ATCCCGAGCAGAGAGCCTAA | GACCCACAGTCACAGGTCAG |
| Sprr3 | CCGTGGTTCCTGGATCATGC | GAAGTACTGGAAGGACTGGGC |
| Cnfn | CAGACACAGCTCAGCGATTG | AAGTCATCGGAGATGCGGC |
| Sprr2f | AACAGCAGTGCAAGCAACCC | CACTTTGGAGGAGGACAAGGCT |
| Myl9 | GGAAGAACCCCACAGACGAG | ATTGCGGATCACATCCTCGG |
| Sorbs1 | ACCAGACAAAGACATGGACCC | ATGCTTCGGAGATTCCTGGG |
| Pde5a | CTGGATGATCACCGGGACTTT | GAGAGCAAGAGCAGGACTCG |
| Nbl1 | TCAGTTACAGCGTCCCCAAC | TCTACCAGCTTGTCCACCCT |
| Bmp3 | ATATCGGCTGGAGCGAATGG | GCTCGCACTATGCTCTGGAT |
| Fam129a | GCTTTCTGCAATCATGTGCG | CTTCAGCAAACTGGGAGAGC |
| Decr2 | CTGTGGATGCTATGACGCGA | GAACTGGCATTGGAGCCTCT |
| Ndrg2 | TGCCAAGGAGGCCGAGTTAG | TGGGGGTGCCATACACAGTA |
| Serping1 | AGTGCCCATGATGAGTAGCG | CACGGGTACCACGATCACAA |
